# *De novo* transcriptomic analysis of cowpea (*Vigna unguiculata* L. Walp*.*) for genic SSR marker development

**DOI:** 10.1186/s12863-017-0531-5

**Published:** 2017-07-11

**Authors:** Honglin Chen, Lixia Wang, Xiaoyan Liu, Liangliang Hu, Suhua Wang, Xuzhen Cheng

**Affiliations:** The National Key Facility for Crop Gene, Resources and Genetic Improvement, Institute of Crop Science, Chinese Academy of Agricultural Sciences, Beijing, 100081 China

**Keywords:** *Vigna unguiculata* (L.) Walp, Transcriptome, *De novo* assembly, Functional annotation, Genic SSR

## Abstract

**Background:**

Cowpea [*Vigna unguiculata* (L.) Walp.] is one of the most important legumes in tropical and semi-arid regions. However, there is relatively little genomic information available for genetic research on and breeding of cowpea. The objectives of this study were to analyse the cowpea transcriptome and develop genic molecular markers for future genetic studies of this genus.

**Results:**

Approximately 54 million high-quality cDNA sequence reads were obtained from cowpea based on Illumina paired-end sequencing technology and were *de novo* assembled to generate 47,899 unigenes with an N50 length of 1534 bp. Sequence similarity analysis revealed 36,289 unigenes (75.8%) with significant similarity to known proteins in the non-redundant (Nr) protein database, 23,471 unigenes (49.0%) with BLAST hits in the Swiss-Prot database, and 20,654 unigenes (43.1%) with high similarity in the Kyoto Encyclopedia of Genes and Genomes (KEGG) database. Further analysis identified 5560 simple sequence repeats (SSRs) as potential genic molecular markers. Validating a random set of 500 SSR markers yielded 54 polymorphic markers among 32 cowpea accessions.

**Conclusions:**

This transcriptomic analysis of cowpea provided a valuable set of genomic data for characterizing genes with important agronomic traits in *Vigna unguiculata* and a new set of genic SSR markers for further genetic studies and breeding in cowpea and related *Vigna* species.

**Electronic supplementary material:**

The online version of this article (doi:10.1186/s12863-017-0531-5) contains supplementary material, which is available to authorized users.

## Background

Cowpea [*Vigna unguiculata* (L.) Walp.] is a diploid *Vigna* crop (2n = 2× = 22), and its genome size is estimated to be 620 Mb [1]. It is the most important legume and semi-arid crop in sub-Saharan Africa and other parts of the world [2, 3] and is widely cultivated in Africa, Latin America, Southeast Asia, and southwestern regions of North America. Its global annual production is approximately 5.8 million tons, with a minimum of 11 million hectares planted [4]. Additionally, cowpea plays an important role in human nutrition and is a critical nutrition source for animals in the dry season in many countries. With its highly efficient nitrogen fixation during crop rotation with cereal crops, this plant can be used to boost soil fertility. Moreover, cowpea is an important income source for farmers and grain traders [5].

Despite its importance, cowpea has received little attention from the research perspective and is less studied than other legumes, such as common bean, chickpea, pea, and soybean. Only a relatively small number of expressed sequence tags (ESTs) and genome sequences of cowpea are available in public databases [1], even though its genome size is relatively small among legume species. The number of microsatellite markers in cowpea is still far fewer than those reported for other legume species, and the genetic base of cowpea is narrow. Informative molecular markers are largely lacking in cowpea. Research on its genome, genetic mapping and molecular breeding has lagged behind those of other pulse crop species, including common bean (*Phaseolus vulgaris*) and soybean (*Glycine. max*). However, significant progress has been made in genomic research on *Medicago truncatula* [6], *Lotus japonicas* [7], soybean (*Glycine max*) [8], pigeonpea (*Cajanus cajan*) [9], chickpea (*Cicer arietinum*) [10], common bean (*Phaseolus vulgaris*) [11], mung bean (*Vigna radiate*) [12], and adzuki bean (*Vigna unguiculata*) [13]. Many ESTs and complete genome sequences of these other legume species are available.

Various molecular markers have been developed in cowpea, such as RFLP (restriction fragment length polymorphism) [14], RAPD (random-amplified polymorphic DNA) [15], and AFLP (amplified fragment length polymorphism) [16]. However, the developed markers are insufficient for an informative genetic study as they are neither highly polymorphic nor easy to analyse. Microsatellite (SSR) markers are usually more informative and have high polymorphism, and they are highly abundant and well distributed throughout the genome in various plants. These SSR markers are a powerful tool for genetic diversity analysis, map construction, quantitative trait locus (QTL) identification, and marker-assisted breeding, and they enhance genetic research in cowpea regarding the genetic improvement of important traits [17]. However, due to a single domestication event [18] and its inherent self-pollination systems [4], cowpea has narrow genetic variability (approximately 16%). The available molecular markers for cowpea are limited, and polymorphic SSR markers in cowpea may be difficult to identify [18]. These issues have been a constraint on cowpea genotyping and other genetic studies. A total of 15 polymorphic SSR markers were obtained from 30 cowpea microsatellite primer pairs [19]. A total of 1071 SSRs were identified in 15,740 cowpea unigene sequences downloaded from the National Center for Biotechnology Information (NCBI), and 102 SSR markers were finally characterized and validated [20]. However, the reported SSR markers in cowpea are not sufficient for an informative genetic study, and more efficient RNA-Seq designs and marker discovery techniques are needed to enhance marker development in the future. Many SSR markers have been developed in common bean [21], chickpea [22], pigeonpea [23], soybean [24], rice bean [25], adzuki bean [26], and mung bean [27]. Thus, there is *an urgent need to develop numerous SSR markers that can be used by breeders in various molecular breeding strategies to increase breeding efficiency* [28].

Transcriptomic data from RNA-Seq allows the efficient identification of large numbers of genic molecular markers compared to the traditional approaches of microsatellite development. Transcriptomic data can also increase the possibility of developing microsatellites associated with functional genes. Previous studies have shown that EST sequences generated using transcriptome techniques are an effective source of SSR markers for legume crops [21, 25–27, 29] and other crops [30, 31]. *De novo* assembly of transcriptome data generated by RNA-Seq will provide valuable genetic resources for further genomic studies and for developing genic SSRs for a crop with a low number of markers available for the species. This study aims to develop more genic SSR markers for cowpea through RNA-Seq technology, characterize the distribution of SSR motifs in the obtained sequences, and validate a set of genic SSR markers.

## Results

### Sequencing and *de novo* assembly

**Fig. 1:**
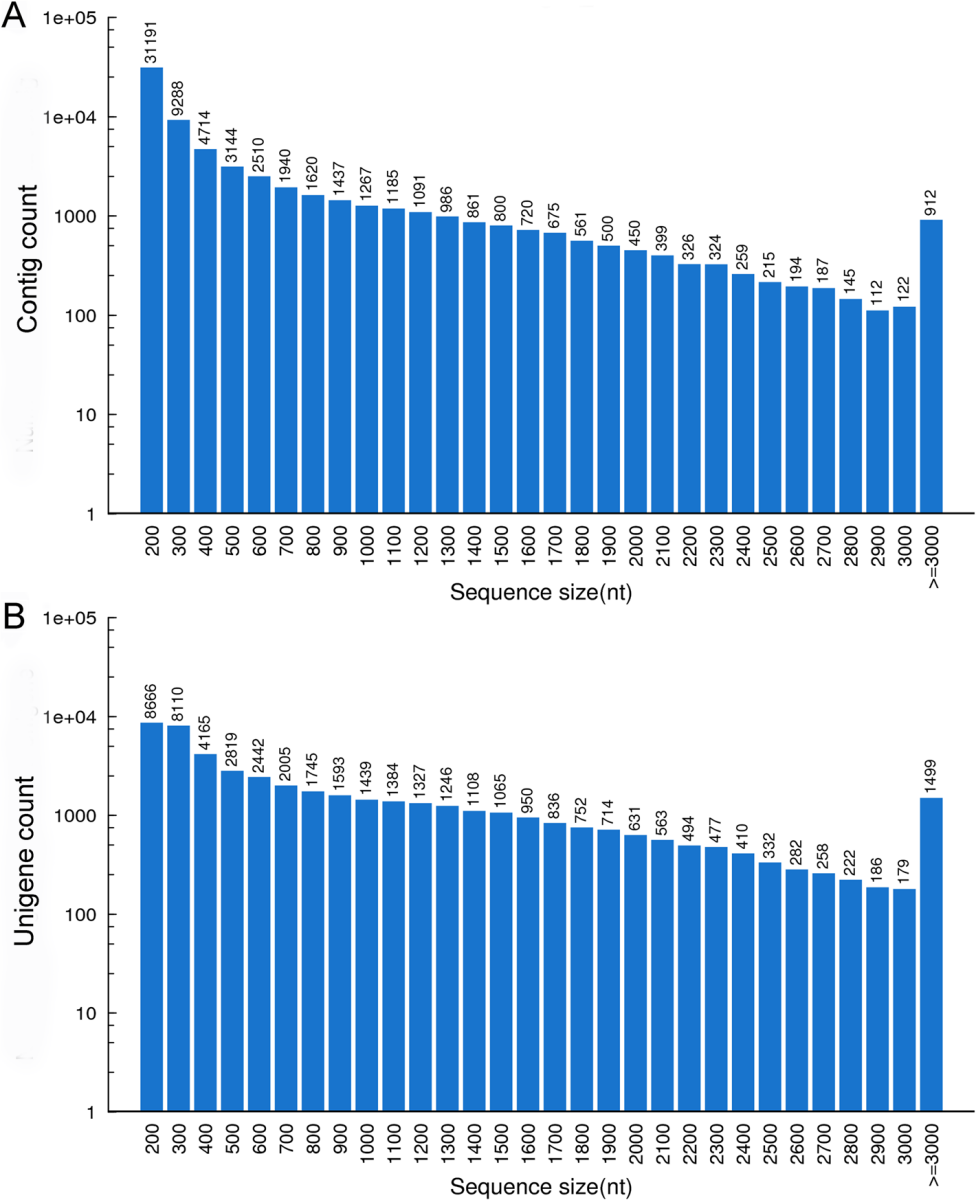
Sequence length distribution of the assembled contigs (**a**) and unigenes (**b**)

A total of 57,214,890 paired-end raw reads were generated by Illumina next-generation sequencing for the cowpea cultivar Zhongjiang No. 1. After quality filtering of the reads, 54,417,074 clean reads were obtained with a GC content of 46.7%, and the Q20 percentage was over 98%. These clean reads were assembled into 68,135 contigs and 47,899 unigenes, and the average length of the assembled unigenes was 871 bp (N50 = 1534 bp). The sequences of the unigenes are listed in Additional file 1. The 68,135 contigs were composed of 48,337 contigs (70.9%) within the length range from 201 to 500 bp, 8774 contigs (12.9%) within the length range from 501 to 1000 bp, 4923 contigs (7.2%) within the length range from 1001 to 1500 bp, 2906 contigs (4.3%) within the length range from 1501 to 2000 bp, 1523 contigs (2.2%) within the length range from 2001 to 2500 bp, and 760 contigs (1.1%) with a length longer than 3000 bp (Fig. 1a). The 47,899 unigenes were composed of 23,760 unigenes (49.6%) within the length range from 201 to 500 bp, 9224 unigenes (19.3%) within the length range from 501 to 1000 bp, 6130 unigenes (12.8%) within the length range from 1001 to 1500 bp, 3883 unigenes (8.1%) within the length range from 1501 to 2000 bp, and 4902 unigenes (10.2%) with a length longer than 2000 bp (Fig. 1b).

### Sequence annotation and functional classification

The *de novo* assembly yielded 47,899 unigenes. Among these unigenes, 36,289 (75.8%) showed high similarity to known proteins in the Nr protein database, while 23,471 unigenes (49.0%) were homologous to proteins in the Swiss-Prot database (Additional file 2). A total of 38,931 unigenes were annotated in the known databases.

**Fig. 2:**
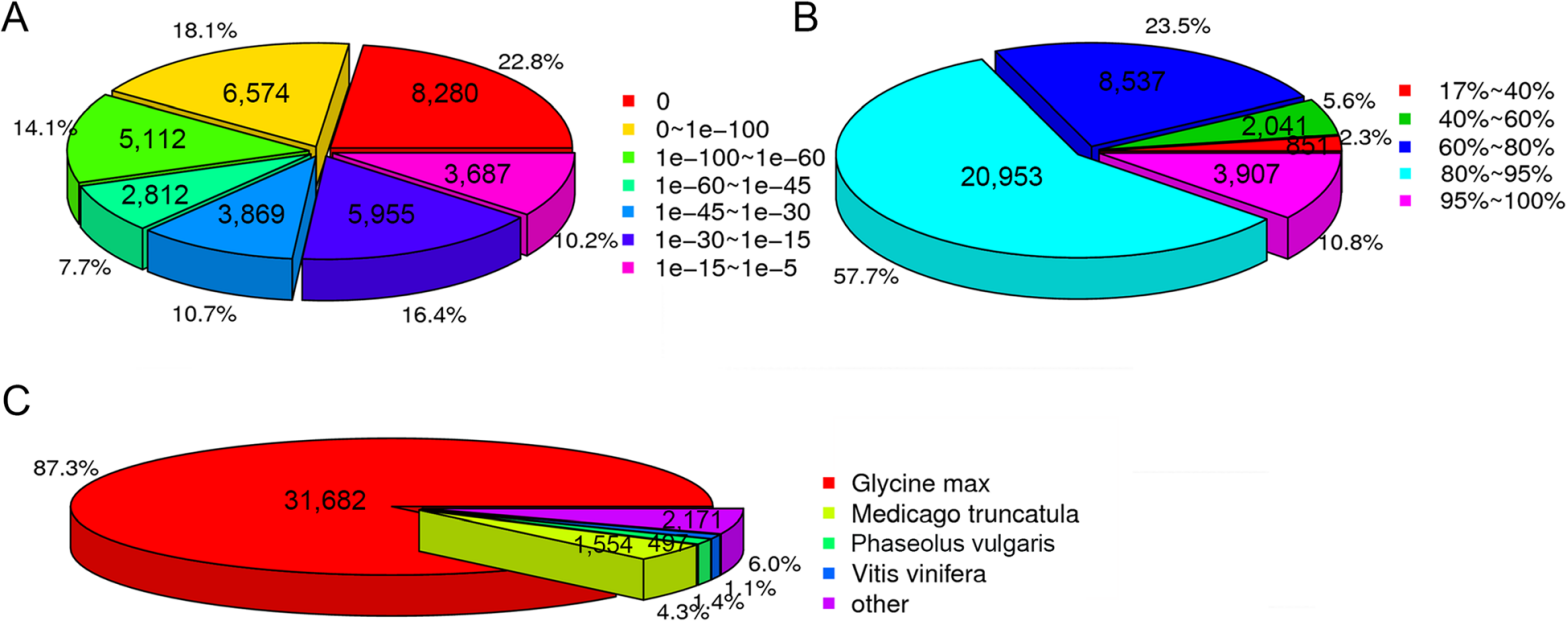
Characteristics of the unigene similarity search against the Nr dataset. **a**: E-value distribution of the BLAST hits for each unigene, with an E-value threshold of 10^−5^ in the Nr database. **b**: Similarity distribution of the top BLAST hits for each unigene in the Nr database. **c**: Species distribution of the top BLAST hits for each unigene in the Nr database

According to the E-value distribution of the unigene hits in the Nr database, 24,860 (68.5%) of the unigenes had a similarity greater than 80% to known plant sequences; 26,647 (73.4%) of the mapped unigenes had high homology (E-value, 1.0E-30) with the available plant sequences; 22,778 (62.8%) of the unigenes had E-values less than 1.0E-45; and 26.6% of the homologous sequences had E-values within the range from 1.0E-5 to 1.0E-30 (Fig. 2a). In the sequence similarity distribution analysis, 851 (2.3%), 2041 (5.6%), 8537 (23.5%), 20,953 (57.7%), and 3907 (10.8%) sequences were 19–40%, 41–60%, 61–80%, 81–95%, and 96–100% similar to those in the Nr database, respectively (Fig. 2b). For the species distribution in Leguminosae, *Glycine max* was ranked first with 31,682 (87.3%) of the top BLASTx hits, followed by *Medicago truncatula*, *Phaseolus vulgaris*, *Vigna unguiculata*, *Vigna radiata*, *Vigna angularis*, *Phaseolus aureus* and *Pisum sativum* with 1554 (4.3%), 497 (1.4%), 258 (0.6%), 80 (0.2%), 44 (0.1%), 26 (0.1%), and 20 (0.1%) hits, respectively.

In addition, 1737 (4.8%) of the sequences matched sequences from other species, including 385 (1.1%) of the sequences in *Vitis vinifera* (Vitaceae), 322 (0.9%) of the sequences in *Lotus corniculatus* var. japonicas (Gramineae), 287 (0.8%) of the sequences in *Amygdalus persica* (Rosaceae), and 71 (0.2%) of the sequences in *Arabidopsis thaliana* (Brassicaceae) (Fig. 2c).

**Fig. 3:**
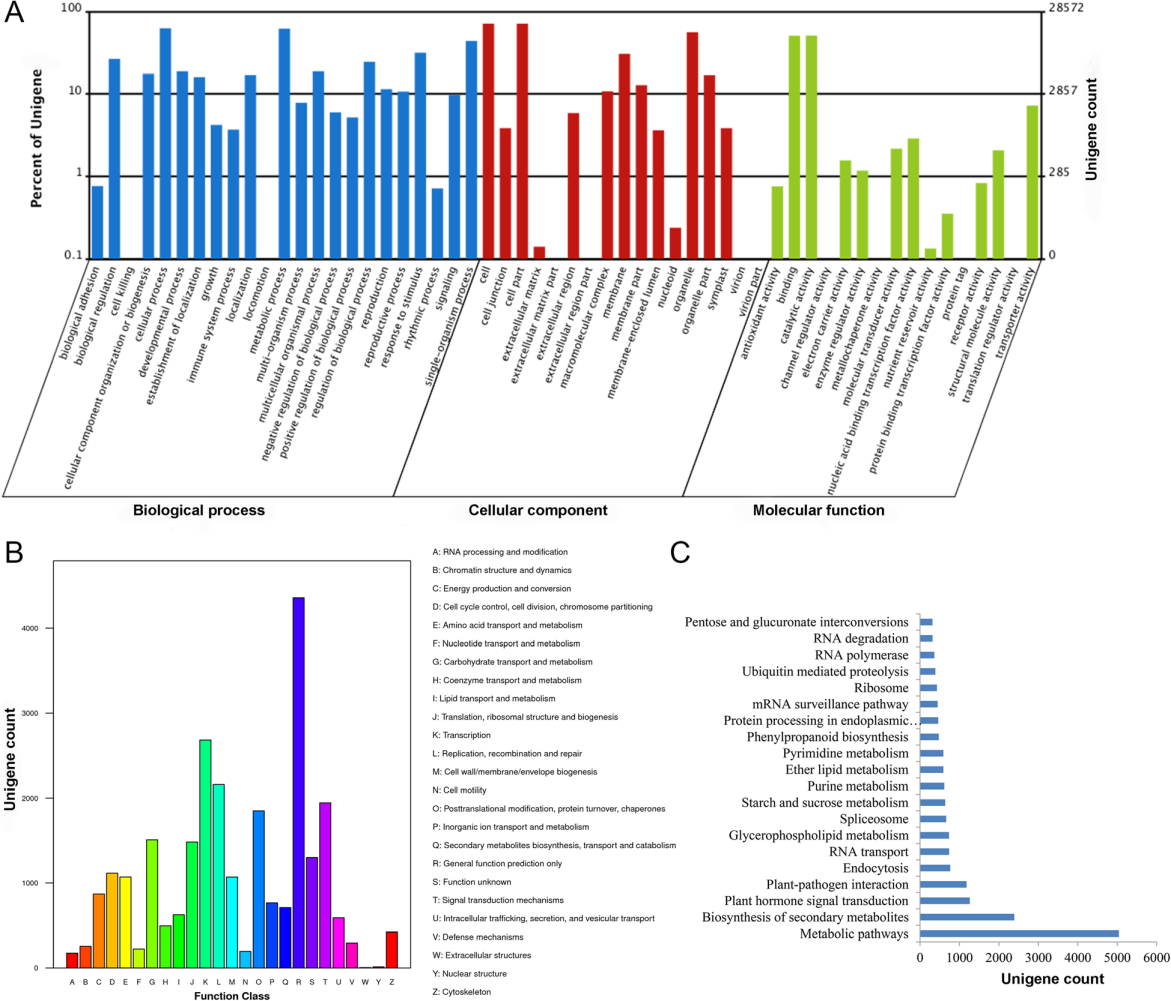
Annotation of the cowpea unigenes. **a**: Gene ontology (GO) annotation of the cowpea unigenes. The results are summarized in three main categories: biological process, cellular component, and molecular function. **b**: COG functional classification of the cowpea unigenes. **c**: Histogram of the KEGG classification of the assembled unigenes in cowpea

The unigenes with GO annotation were classified into three categories: biological processes, cellular components, and molecular functions (Fig. 3a). Among them, unigenes for biological processes accounted for the majority of the unigenes (113,804, 49.4%), followed by unigenes for cellular components (81,937, 35.6%) and unigenes for molecular function (34,482, 15.0%), covering a comprehensive range of GO categories. Among the biological process categories, unigenes of the cellular (17,837, 15.7%), metabolic (17,625, 15.5%), and single-organism (12,493, 11.0%) processes accounted for the majority of the unigenes. However, only a few unigenes were assigned to the categories of locomotion (25) and cell killing (2). In the category of cellular components, unigenes of cell (20,309, 34.8%) and organelle (15,969, 19.5%) components were the dominant unigenes, whereas only a few unigenes were involved in the extracellular matrix (17) and virion (7) components. In the molecular function category, catalytic activity (14,532, 42.1%) and binding (14,479, 42.0%) were the two main categories. Moreover, 2066 unigenes were assigned to transporter activity, whereas only a few unigenes were involved in translation regulator (3), protein tag (2), and channel regulator (1) activity.

According to Cluster of Orthologous Groups of proteins (COG) functional classification, 26,170 (30.8%) unigenes were aligned to the COG database and categorized into 25 functional categories (Fig. 3b); among these categories, the general function group represented the largest group (4358 genes, 16.7%), followed by the transcription (2683 genes, 10.3%); replication, recombination, and repair (2158 genes, 8.3%); signal transduction mechanism (1945 genes, 7.4%); and posttranslational modification, chaperones, and protein turnover (1850 genes, 7.1%) groups. Nuclear structures (13 genes) and extracellular structures (5 genes) represented the smallest groups predicted by COG.

The pathway annotations of the unigene sequences were analysed using the KEGG mapper. A total of 20,654 unigenes were assigned to 5 major categories in 128 pathways that showed high similarity (Fig. 3c). Among these unigenes, metabolic pathways contained 5040 (24.4%) unigenes, followed by biosynthesis of secondary metabolites (2390, 11.6%), plant hormone signal transduction (1262, 6.1%), plant-pathogen interaction (1179, 5.7%), and endocytosis (765, 3.7%).

### Frequency and distribution of genic SSRs

Among the 47,899 unigenes found in this study, 4729 (9.9%) unigenes contained one or more SSR sequences. Additionally, 690 (1.4%) unigenes contained at least two independent SSR sequences, and 235 (0.5%) contained compound SSRs of different motifs. These compound SSRs were not evenly distributed in various genic SSR units or groups. Tri-nucleotide (2500), di-nucleotide (1540), mono-nucleotide (1164), hexa-nucleotide (155), penta-nucleotide (137), and tetra- nucleotide (64) motifs accounted for 45.0%, 27.7%, 20.9%, 2.8%, 2.5%, and 1.2%, respectively; the tri-nucleotide motifs (2500, 45.0%) were the most abundant, followed by the di-nucleotide motifs (1540, 27.7%) (Table 1).

**Table 1:** Summary of the numbers of repeat units in cowpea genic SSR loci

Repeat motif	No. of repeats								
	4	5	6	7	8	9	10	>10	Total
Mono-nucleotide (1164)									
A/T	-	-	-	-	-	-	-	1161	1161
C/G	-	-	-	-	-	-	-	3	3
Di-nucleotide (1540)									
AG/CT	-	-	418	198	150	125	134	71	1096
AT/AT	-	-	91	51	41	12	22	28	245
AC/GT	-	-	96	47	28	13	8	7	199
Others	-	-	-	-	-	-	-	-	-
Tri-nucleotide (2500)									
AAG/CTT	-	397	237	137	6	-	-	-	777
ATC/ATG	-	253	102	79	7	-	-	-	441
ACC/GGT	-	174	65	32	5	-	-	-	276
AGC/CTG	-	156	43	29	7	-	-	-	235
AAC/GTT	-	142	61	20	3	-	-	-	226
Others	-	337	133	54	21	-	-	-	545
Tetra-nucleotide (64)									
AAAG/CTTT	-	13	2	-	-	-	-	-	15
ACTC/AGTG	-	7	7	-	-	-	-	-	14
AAAT/ATTT	-	10	-	-	-	-	-	-	10
AATG/ATTC	-	8	1	-	-	-	-	-	9
Others	-	12	4	-	-	-	-	-	16
Penta-nucleotide (137)									
AAGAG/CTCTT	38	-	-	-	-	-	-	-	38
AAAAT/ATTTT	13	-	-	-	-	-	-	-	13
AAAAG/CTTTT	10	1	-	-	-	-	-	-	11
AACAC/GTGTT	9	-	-	-	-	-	-	-	9
Others	62	4	-	-	-	-	-	-	66
Hexa-nucleotide (155)									
AAACCC/GGGTTT	7	-	-	-	-	-	-	-	7
AATGGC/ATTGCC	7	-	-	-	-	-	-	-	7
AAAGAG/CTCTTT	6	-	-	-	-	-	-	-	6
AACTTG/AAGTTC	5	-	-	-	-	-	-	-	5
ACAGCC/CTGTGG	5	-	-	-	-	-	-	-	5
Others	125	-	-	-	-	-	-	-	125
Total	287	1514	1260	647	268	150	164	1270	5560
%	5.2	27.2	22.7	11.6	4.8	2.7	3.0	22.8	

The number of SSR repeats per locus ranged from 4 to 24, and SSRs with five repeats were the most abundant, followed by SSRs with six, seven, and twelve random repeats. Motifs with more than 17 repeats accounted for only 6.5% of the total. The (A/T)_n_ repeats were the most abundant (99.7% in mono-nucleotide motif) of the nucleotide repeats. The other six main motif types included the (AG/CT)_n_ di-nucleotide repeat (71.2%), (AAG/CTT)_n_ tri-nucleotide repeat (31.1%), (AAGAG/CTCTT)_n_ penta-nucleotide repeat (27.7%), (AAAG/CTTT)_n_ tetra-nucleotide repeat (23.4%), and (AAACCC/GGGTTT)_n_ hexa-nucleotide repeat (4.5%).

### Development of genic SSR markers

A total of 5560 genic SSR markers were obtained from the 4729 SSR-containing sequences (Additional file 3). To validate their polymorphisms for 32 cowpea accessions, one set of 500 markers was randomly chosen from the above list of loci (Additional file 4). Among the tested markers, 235 primer pairs (47.0%) produced clear amplicons with the expected sizes in nearly all of the 32 cowpea accessions, 73 primer pairs produced non-specific products, and 192 primer pairs produced no clear PCR bands. Among the successful markers, 54 (10.8%) polymorphic genic SSR markers were identified, containing 7 di-, 29 tri-, 2 tetra-, 3 penta- and 13 hexa-motifs, while the other 181 primer pairs were monomorphic.

### Validation of genic SSR markers

**Fig. 4:**
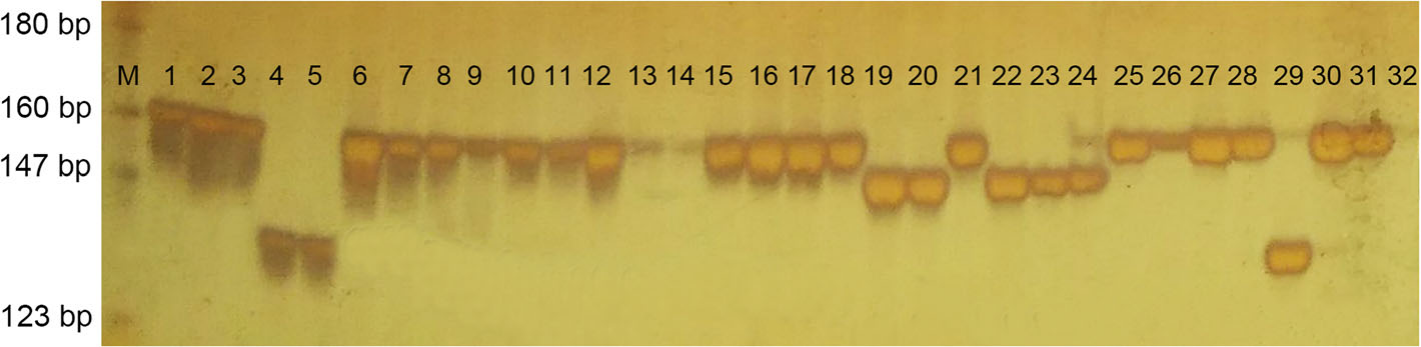
An illustration of a PAGE gel showing allelic variation for the genic SSR marker Vu6289 among 32 cowpea accessions. The 32 cowpea germplasm accessions are listed in Additional file 5. M: pBR322 DNA/MspI DNA ladder

To confirm the 54 polymorphic genic SSRs developed in this study, we performed an extra validation in 32 diverse cowpea accessions (Additional file 5). The electrophoretic profiles of 32 cowpea accessions using marker Vu6289 are shown in Fig. 4. A total of 154 alleles were detected and scored (Table 2). The observed heterozygosity (*Ho*) varied from 0.1316 (Unigene 6390) to 0.9345 (Unigene 6224), and the average was 0.5474. The polymorphic information content (*PIC*) values ranged from 0.0624 (Unigene 6224) to 1.6215 (Unigene 6857), and the average was 0.8392.

**Table 2:** Characteristics of the 54 polymorphic SSR markers validated in 32 different cowpea accessions

Gene locus	*Na*	*Ho*	*PIC*
Unigene4200	2	0.1316	0.4113
CL2002Contig1	2	0.5192	0.3457
Unigene4708	2	0.5023	0.0688
CL4898Contig2	2	0.7506	0.3648
CL4580Contig2	2	0.4918	0.4188
CL150Contig2	2	0.8169	0.3648
Unigene4211	3	0.4289	0.2149
Unigene16847	3	0.4644	0.2688
CL275Contig1	3	0.5277	0.2205
Unigene11213	3	0.4604	0.3648
Unigene11595	2	0.6362	0.2392
Unigene10812	2	0.9273	0.3236
CL2728Contig2	3	0.4364	0.2688
Unigene13454	2	0.5119	0.1638
Unigene5616	2	0.5119	0.3285
Unigene8092	2	0.5480	0.3739
Unigene3540	2	0.5783	0.2938
CL2572Contig1	2	0.5006	0.3833
Unigene9045	2	0.6746	0.3690
Unigene15775	2	0.6746	0.4605
Unigene5773	2	0.7175	0.1638
Unigene15736	2	0.5119	0.3610
CL2115Contig1	3	0.4797	0.3747
Unigene17353	2	0.8169	0.4634
Unigene10711	2	0.4938	0.3705
Unigene14297	2	0.5692	0.4217
CL2511Contig5	2	0.7428	0.3318
Unigene6203	3	0.3682	0.5556
Unigene6211	3	0.4156	0.5193
Unigene6270	3	0.4328	0.4956
Unigene6222	2	0.7013	0.2503
Unigene6346	4	0.4311	0.4631
Unigene6311	2	0.5402	0.3498
Unigene6298	3	0.5684	0.3833
Unigene6357	6	0.1972	0.7588
Unigene6375	2	0.7096	0.2447
Unigene6277	6	0.3533	0.5969
Unigene6300	2	0.8734	0.1167
Unigene6258	4	0.4789	0.4777
Unigene6224	2	0.9345	0.0624
Unigene6249	3	0.8064	0.1807
Unigene6195	4	0.6277	0.1787
Unigene6192	4	0.4153	0.3410
Unigene6218	2	0.8169	0.4641
Unigene6327	3	0.4645	0.1638
Unigene6333	2	0.8169	0.1545
Unigene6683	7	0.1949	0.4690
Unigene6214	2	0.6746	0.1638
Unigene6265	5	0.2329	0.7621
Unigene6390	10	0.1316	0.2688
Unigene6770	3	0.4492	0.7172
Unigene6857	2	0.4973	0.8392
Unigene6347	3	0.3254	0.4598
Unigene6213	2	0.3124	0.3153
Mean	2.8	0.5474	0.3440
St.	1.5	0.1926	0.1476

Number of alleles (*Na*), observed heterozygosity (*Ho*) and polymorphic information content (*PIC*)

These unigene sequences of 54 polymorphic genic SSRs were subjected to BLASTn analysis, and all the sequences were similar to protein-encoding genes from mung bean (*Vigna radiata*) and adzuki bean (*Vigna angularis*). A few examples were genes for the auxin-responsive protein (IAA16), E3 ubiquitin protein ligase (DRIP2), ABC transporter, phosphomethylethanolamine N-methyltransferase, leucine zipper protein (ATHB-14), dof zinc finger protein (DOF1.8), ethylene-responsive transcription factor (TINY) and glycine-rich cell wall structural protein (Additional file 6).

## Discussion

Our transcriptomic analysis revealed a comprehensive transcriptome of the cowpea using Illumina sequencing technology, contributed a new and significant set of 54 million high-quality cDNA sequence reads, and provided a set of 47,899 unigenes for cowpea. Our analysis also identified 5560 genic SSRs; this is the first large-scale development of SSR markers in cowpea. These findings not only provide valuable information for genetic diversity, linkage mapping, gene/QTL mapping, and marker-assisted selection in cowpea but also show promise in the search for informative molecular markers in a crop plant with few genomic resources.

Compared with genomic SSRs, genic SSRs, being parts of genes, are more useful as molecular markers, as they represent variation in the expressed part of the genome. Additionally, genic SSR-flanking sequences are highly conserved and show high transferability to related species or genera owing to the higher conservation of expressed sequences across species. Genic SSR markers developed in one species could be used in related species or genera for which sufficient sequence information is not available for marker development [32]. Previously, genome conservation among different legume genera was detected with DNA markers [19, 33–36]. Cowpea showed the greatest similarity with common bean, mung bean, adzuki bean, rice bean and black gram. High degrees of conservation and synteny among these five legumes were revealed through comparative mapping [37, 38]. This may reflect the genetic relatedness and higher gene homology among these five genomes, as all are self-pollinated diploid legumes (2n = 2× = 22) with a similar genome size and belong to the genus *Vigna*.

Genic SSRs have also been applied to analyse genetic diversity, to map QTLs controlling agronomically important traits and to increase the efficiency of marker-assisted selection [32]. However, traditional Sanger sequencing of cDNAs could not provide enough unigenes or sufficient contig lengths because of the limitation of the sequenced length even if full-length cDNA libraries were adopted [39]. Transcriptomic data from RNA-Seq are useful for analysing transcriptome sequencing and assembling in many plants. This technique is rapid and cost-effective for developing microsatellite markers [21, 25] and specifically is a promising method for marker analysis in species without sequenced genomes. Our analysis has shown the usefulness of transcriptome sequencing in unigene assembly and marker development in cowpea.

In the present study, a total of 54.42 million clean reads with a length of 4,897,536,660 bp were obtained using Illumina paired-end sequencing technology. Additionally, 98.45% of the clean reads had Phred quality scores at the Q20 level, and the percentage of ambiguous “N” bases was 0.01%, ensuring the quality of the sequencing. Furthermore, 47,899 unigenes were assembled from the cowpea transcriptome, with an average unigene length of 871 bp, which is similar to other *Vigna* plants, as reported in mung bean (874 bp) [40]. The average unigene length was longer than that of black gram (443 bp) [41] but was shorter than that reported in other *Vigna* plants such as rice bean (986 bp) [25] and adzuki bean (1213 bp) [26]. This difference may reflect a difference of species and of the assembler and parameters; for example, a longer mean length of unigenes in adzuki bean is due to the well-assembled reference genome of adzuki bean.

As a preliminary study, a total of 1071 SSRs in 15,740 cowpea unigene sequences were identified; as a result, 102 SSR markers and 1536 SNP markers were developed [20]. Genic SSRs have been broadly developed in many legume species, including common bean [21], chickpea [22], pigeonpea [23], rice bean [25], adzuki bean [26], mung bean [40], and black gram [41]. A total of 3011, 7947, 13,134 and 1840 genic SSRs were identified from 71,929 rice bean unigenes [24], 65,950 adzuki bean unigenes [25], 48,693 mung bean unigenes [40] and 48,291 black gram transcript contigs [41], respectively. In this study, a total of 5560 genic SSR markers were developed from 4729 SSR-containing unigene sequences. To the best of our knowledge, this is the first large-scale development of SSR markers in cowpea. The new SSR sequences and genic SSR markers provide not only important genomic resources for basic research but also some new opportunities to find closely linked markers for traits of agronomic importance for the genetic improvement of cowpea.

To determine the polymorphism levels of the developed genic SSR markers, we evaluated 500 genic SSR loci, and 235 primer pairs (47.0%) produced successful amplicons. Additionally, 54 new polymorphic genic SSR markers were characterized and validated. A low polymorphism level was detected in 32 cowpea accessions, and the valid primer ratio was only 10.8% (54 in 500), which is lower than that from previous reports in other related legume species, including mung bean (33%) [40], black gram (58.2%) [41], common bean (71.3%) [42] and chickpea (47.3%) [43] but higher than that reported in rice bean (7.6%) [25] and adzuki bean (7.6%) [26]. The ratio of effective to non-effective primer design in these results was higher than that reported from BAC-derived SSRs in common bean [44] or from the screening of non-enriched small-insert genomic libraries [45]. The location of primers across splice sites or regions of poor sequence quality could explain the non-amplification. More than two-thirds of the genic SSR markers produced successful amplicons, suggesting that the quality of our assembled unigenes is high.

In this study, the tri-nucleotide motifs (2500, 20.9%) and di-nucleotide motifs (1540, 27.7%) were the most abundant. The types of motifs found in SSR loci were similar to the previous results on microsatellites in plants [46]. A relatively large proportion of the di- and tri-nucleotide repeats in EST sequences has been reported in common bean [21] and chickpea [22]. The most common tri-nucleotide repeat found in cowpea was AAG/CTT, followed by ACC/GGT and AAC/GTT. The most common di-nucleotide repeat was AG/CT, followed by AT/AT and AC/GT. The results are similar to previous studies in adzuki bean [47], common bean [48], and fava bean [49]. Additionally, the number of alleles in most of the successfully amplified SSRs was less than four. The PIC values in the current study ranged from 0.0624 to 1.6215, with an average of 0.8392, which is in line with previous reports for cowpea [20] and mung bean [40] SSRs.

Applicable microsatellite loci were identified, and some of them may be useful in genetic research. However, there are some limitations in our study. The cDNA library was established using only one cowpea variety, which may cause an ascertainment bias. Second, only 500 of 5560 SSR markers were randomly selected for further validation, limiting their application potential. Third, more efficient RNA-Seq designs are needed to enhance marker development in transcriptomic studies. Fourth, the polymorphism rate is relatively low. Such a low rate of polymorphisms may be due to (i) a large intron between primers that would prevent amplification of the genomic sequence based on the transcribed mRNA sequence, (ii) the priming sites being disrupted by unrecognized intron splice sites, and (iii) low-quality sequences, fragmented assembly or assembly errors. The failure of 24 primer pairs to generate amplicons might be caused by long intervening introns, which would prevent PCR amplification of selected markers.

## Conclusions

The transcriptomic analysis in our study was fruitful, providing not only a valuable set of genomic data characterizing genes for important agronomic traits in *Vigna unguiculata* but also a new set of genic SSR markers for further genetic studies and breeding in cowpea and related *Vigna* species. Our analysis also showed the promise of using the RNA-Seq technique for SSR development in crop plants, such as cowpea, with few or no genomic resources.

## Methods

### Plant materials

A set of 32 Chinese cowpea accessions was used in SSR marker testing, which included 14 accessions from Hunan province, 9 accessions from Hubei province, and 9 accessions from Anhui province. These cowpea germplasm accessions were obtained from the National Gene Bank (China, Beijing). The cowpea seedlings were grown in plastic pots (20 cm × 15 cm × 15 cm) under a 14/10 h photoperiod at 25 °C (day) and 20 °C (night) in a greenhouse at the Institute of Crop Science, Chinese Academy of Agricultural Sciences (China, Beijing, 116°46′E, 39°92′N). At the three-week-old seedling stage, five plants of “Zhongjiang No. 1” were transferred to a programmable incubator for dehydration for 3 h at 28 °C, termed drought stress, and the seedlings were then harvested, frozen immediately in liquid nitrogen, and stored at −80 °C for RNA-Seq.

### RNA extraction

Total RNA was isolated from three different tissues, including roots, stems, and leaves, of five cowpea seedlings, and these RNA samples were mixed according to the equivalent concentration of these RNA samples were mixed, and 20 μg of RNA was quickly frozen in liquid nitrogen and then stored at −80 °C. The RNA was isolated with TRIZOL reagent (Invitrogen, Carlsbad, CA, USA) following the manufacturer’s instructions. The residual DNA was removed by incubation with RNase-free DNase I (TaKaRa, Kyoto, Japan) for 30 min at 37 °C. The concentration and integrity of the total RNA was measured by a 2100 Bioanalyzer (Agilent, Santa Clara, CA) at 260 nm and 280 nm and checked for degradation by RNase-free agarose gel electrophoresis. The RNA was further quantified with an RNA Nanochip. More than 20 μg of total RNA (concentration ≥ 500 ng/μL, OD260/280 = 1.8–2.2, OD260/230 ≥ 2.0, and RNA integrity number (RIN) ≥ 8.0) was used to create the full-length cDNA library.

### cDNA library establishment and sequencing

The cDNA library was constructed using the NEBNext Ultra RNA Library Prep Kit (NEB, Ipswich, USA) according to the manufacturer’s instructions [50]. The total RNA was used to isolate poly(A) RNA using poly-T oligo-attached magnetic beads (Illumina, San Diego, CA, USA). The poly(A) RNA was then fragmented into smaller pieces using divalent cations at 94 °C for 4 min. First-strand cDNA was synthesized using SuperScript II reverse transcriptase and random primers. Second-strand cDNA was synthesized by adding GEX second strand buffer, dNTPs, RNase H, and DNA polymerase I. The cDNA fragments were further subjected to end repair and phosphorylation with T_4_ DNA polymerase, Klenow DNA polymerase, and T_4_ polynucleotide kinase. The repaired cDNA fragments were then 3′-end adenylated with Klenow Exo and ligated with PE adapters. The products of this ligation reaction were purified through 2% (*w*/*v*) TAE-agarose gel, and templates of different sizes were selected for downstream enrichment. The cDNA fragments (200 ± 25 bp) were finally excised from the gel and amplified via PCR to enrich the cDNA template. Purified cDNA templates were enriched by 15 cycles of PCR amplification using PE1.0 and PE2.0 primers and Phusion DNA Polymerase (NEB, USA). The cDNA library was constructed and sequenced in one lane on the Illumina Hiseq™ 2500 platform (Novogene, Beijing, China) with Illumina paired-end sequencing technology, and the mean fragment length was 200 bp (±25 bp) [51]. Finally, the raw data for all the reads were collected from the Illumina system. The raw sequences were submitted to the sequence read archive (SRA) at the National Center for Biotechnology Information (NCBI) under the submission IDs SRR4897234 and SRX2323067 and study accession SRP092517.

### Data filtering and *de novo* assembly

Prior to transcriptome assembly, a stringent filtering process was carried out on the raw sequencing reads. Raw reads in fastq format were initially processed using in-house Perl scripts [51]. All reads with more than 10% of bases having a poor quality score (Q < 20) or non-coding RNA, and ambiguous sequences containing an excess of N nucleotides, were removed by Seqclean. The reads were preprocessed to eliminate low-quality reads (reads with ambiguous “N” bases >5% and more than 10% Q < 20 bases). Reads were discarded that failed to pass the failed-chastity filter based on the relation “failed-chastity ≤1”. In the first 25 cycles, the chastity threshold was less than 0.6 for filtering. In this step, clean data were obtained by removal of adapter-containing, poly-N-containing and low-quality reads from the raw data. Meanwhile, Q20, Q30, and the GC content of the clean data were calculated. All downstream analyses were based on the high-quality clean data. A Perl pipeline was used for analysing sequence data, and *de novo* transcriptome assembly was performed using the program Trinity [52]. The Trinity assembler was used with the inchworm k-mer method, and all the server resources were set to unlimited. The sequences without redundancy, containing the fewest Ns and not being extended on either end, could be defined as unigenes.

### Unigene annotation and classification

The annotation of unigenes was carried out with various bioinformatics procedures. First, these unigenes were used to search various protein databases including Nr, Swiss-Prot, COG, and KEGG (E-value ≤1.0E-5), retrieving proteins with the highest sequence similarity annotated to each unigene. Second, with nucleotide-based annotation, Blast2GO was used to obtain GO annotation categories according to molecular function, biological process and cellular component ontologies [53]. Third, ESTs were annotated into KEGG pathways with the KEGG Automatic Annotation Server (KAAS) [54] using the single-directional best hit (SBH) method. The unigene sequences were also aligned to the COG database to classify and predict their possible functions.

### SSR search and primer design

To amplify a sequence with appropriate length (>100 bp) based on ensuring the quality of the primer, only sequences for which both ends of the SSR had lengths greater than 150 bp were used to design these primers. In this study, the minimum number of repeats for different SSR markers was chosen as follows: eleven for mono-nucleotide, six for di-nucleotide, five for tri- and tetra-nucleotide, and four for penta- and hexa-nucleotide motifs. Primers were designed based on the flanking sequences of these SSRs using Primer Premier 3.0 (http://sourceforge.net/projects/primer3) under the following criteria: primer length 18–22 bp (optimally 20 bp), Tm of 50–60 °C (no more than a 4 °C difference between the annealing temperatures of the forward and reverse primers), and PCR product length in the range of 100–300 bp. Primers were synthesized by Qingke Biotech (Beijing, China).

### Genic SSR characterization and validation

The identified SSR markers were amplified and validated with 32 accessions of cowpea, including fourteen accessions from Hunan Province, nine accessions from Hubei Province and nine accessions from Anhui Province (Additional file 5). These cowpea accessions were preserved in the National Gene Bank at the Institute of Crop Science, Chinese Academy of Agricultural Sciences (Beijing, China, 116°46′E, 39°92′N). Genomic DNA was extracted from young leaves of five plants for each accession using the cetyl trimethyl ammonium bromide (CTAB) method [23]. DNA quality and quantity were analysed by 1% agarose gel electrophoresis. PCR was performed in a system of 20 μL containing 0.5 U of Taq polymerase, 1× PCR Buffer, 1.5 mM MgCl_2_, 25 μM dNTP (Qingke Biotech, Beijing, China), 0.4 μM of each primer, and 30 ng of genomic DNA. PCR was carried out on a Bio-Rad T100™ Thermal Cycler (Bio-Rad, USA). PCR amplification was performed as follows: 94 °C for 4 min and 30 cycles of 94 °C for 30 s, 55 °C for 30 s and 72 °C for 30 s. The final extension was carried out at 72 °C for 5 min. The PCR products obtained were verified in 6.0% non-denaturing PAGE (polyacrylamide gel electrophoresis) using the silver staining method. Fragment sizes were estimated with the pBR322 DNA/MspI DNA ladder (Kangwei Biotech, Beijing, China). To determine the functions of the polymorphic genic SSRs, the unigene sequences used for the development of these markers were subjected to a BLASTn analysis against a non-redundant database of legume sequences.

## Additional files

Additional file 1: Sequences of all unigenes. (XLS 4765 kb)

Additional file 2: Unigenes that were annotated in the Nr database. (XLS 7296 kb)

Additional file 3: A list of 5560 cowpea genic SSR primers developed in this study. (XLS 764 kb)

Additional file 4: Characteristics of 500 primer pairs derived from cowpea genic SSRs. (XLS 199 kb)

Additional file 5: A list of 32 cowpea germplasm accessions used in this study. (DOC 69 kb)

Additional file 6: The putative proteins identified by BLASTX of 54 unigene sequences containing polymorphic genic SSRs. (XLS 35 kb)

## Abbreviations

EST: Expressed sequence tag
Ho: Observed heterozygosity
Na: Alleles
PIC: Polymorphic information content
SSR: Simple sequence repeat
