# Supplementary material for: De novo transcriptomic analysis of cowpea (Vigna unguiculata L. Walp.) for genic SSR marker development
Source: BMC Genet. 2017 Jul 11;18:65. doi: 10.1186/s12863-017-0531-5 (PMC5504845; doi:10.1186/s12863-017-0531-5)
Supplement: Supplementary file 5 — A list of 32 cowpea germplasm accessions used in this study. (DOC 69 kb) [file 12863_2017_531_MOESM5_ESM.doc]

**Additional file 5:** A list of 32 cowpea germplasm accessions used in this study

| Serial No. | Accession No. | Province of origin | Seed pattern | Plant habit |
| --- | --- | --- | --- | --- |
| 1 | I0003383 | Hunan | Brown | Semiprostrate |
| 2 | I0003411 | Hunan | White | Semiprostrate |
| 3 | I0003385 | Hunan | Brown | Prostrate |
| 4 | I0003430 | Hunan | Brown | Erect |
| 5 | I0003421 | Hunan | Brown | Prostrate |
| 6 | I0003414 | Hunan | Brown | Prostrate |
| 7 | I0003419 | Hunan | Brown | Prostrate |
| 8 | I0003408 | Hunan | White | Semiprostrate |
| 9 | I0003422 | Hunan | Pink | Prostrate |
| 10 | I0003437 | Hunan | Red | Prostrate |
| 11 | I0003407 | Hunan | White | Prostrate |
| 12 | I0003428 | Hunan | Variable | Prostrate |
| 13 | I0003416 | Hunan | White | Prostrate |
| 14 | I0003418 | Hunan | Brown | Erect |
| 15 | I0000225 | Hubei | Variable | Prostrate |
| 16 | I0002075 | Hubei | Pink | Prostrate |
| 17 | I0002096 | Hubei | Black | Prostrate |
| 18 | I0002251 | Hubei | Pink | Prostrate |
| 19 | I0000216 | Hubei | Pink | Semiprostrate |
| 20 | I0002534 | Hubei | Red | Prostrate |
| 21 | I0002473 | Hubei | Brown | Prostrate |
| 22 | I0000792 | Hubei | Pink | Prostrate |
| 23 | I0000768 | Hubei | Pink | Prostrate |
| 24 | I0001251 | [Anhui](javascript:void(0);) | Brown | Prostrate |
| 25 | I0001183 | [Anhui](javascript:void(0);) | Pink | Semiprostrate |
| 26 | I0001748 | [Anhui](javascript:void(0);) | Pink | Prostrate |
| 27 | I0001736 | [Anhui](javascript:void(0);) | Brown | Prostrate |
| 28 | I0001194 | [Anhui](javascript:void(0);) | Brown | Prostrate |
| 29 | I0001757 | [Anhui](javascript:void(0);) | Brown | Semiprostrate |
| 30 | I0001735 | [Anhui](javascript:void(0);) | White | Semiprostrate |
| 31 | I0001753 | [Anhui](javascript:void(0);) | Brown | Semiprostrate |
| 32 | I0001742 | [Anhui](javascript:void(0);) | Pink | Semiprostrate |

These accessions were from National Center for Crop Germplasm Resources Preservation of China.
